# Does Non-surgical Periodontal Therapy With Adjunct Antimicrobial Photodynamic Therapy Help Reduce Periodontal Inflammation and Haemoglobin A1c Levels in Patients With Type-2 Diabetes Mellitus? A Systematic Review and Meta-analysis

**DOI:** 10.3290/j.ohpd.b5750814

**Published:** 2024-09-17

**Authors:** Munerah S. BinShabaib

**Affiliations:** a Department of Preventive Dental Sciences, College of Dentistry, Princess Nourah Bint Abdulrahman University, Riyadh, Saudi Arabia

**Keywords:** diabetes mellitus, haemoglobin A1c, non-surgical periodontal therapy, periodontal disease, photodynamic therapy

## Abstract

**Purpose:**

The aim of the present systematic review and meta-analysis was to assess the efficacy of non-surgical periodontal therapy (NSPT) with adjunct photodynamic therapy (aPDT) in reducing periodontal inflammation and haemoglobin A1c (HbA1c) levels in patients with diabetes mellitus (DM).

**Materials and Methods:**

The focused question was ‘Does NSPT with adjunct aPDT help reduce periodontal inflammation and HbA1c levels in patients with DM?’ The PICO (patient/population, intervention, comparison and outcomes) was formatted as follows: Patients (P): Participants diagnosed with DM; Intervention (I): NSPT with adjunct PDT for the treatment of periodontitis; Control (C): NSPT alone or NSPT with adjunct systemic antibiotic therapy; and Outcome (O): Changes in HbA1c levels. The inclusion criteria comprised RCTs specifically evaluating the impact of NSPT on HbA1c levels in diabetic patients, with a specific focus on interventions involving NSPT with and without adjunct aPDT. The literature search was performed in accordance with the Preferred reporting items for systematic reviews and meta-analysis. Indexed databases were searched without time and language restrictions using various keywords. Forest plots were created to illustrate the effects of the different studies and the global estimation.

**Results:**

Five RCTs were included and processed for data extraction. The number of participants ranged from 12 to 45 patients with medically diagnosed type-2 DM. In all RCTs, aPDT was done using a diode laser with wavelengths ranging between 660 and 810 nm. Three and two RCTs had moderate and high RoB, respectively. In two RCTs, NSPT with adjunct aPDT reported no improvement in clinical periodontal parameters. Two studies reported that NSPT with adjunct aPDT significantly reduces periodontal probing depth compared to NSPT alone. Four of the five RCTs reported that NSPT+PDT does not influence HbA1c levels.

**Conclusions:**

NSPT with or without adjunct aPDT does not affect HbA1c levels in patients with type-2 DM.

Non-surgical periodontal therapy (NSPT), also known as scaling and root planing (SRP), is a fundamental component of the comprehensive management of periodontal diseases such as periodontitis.^11^ This non-invasive approach is designed to eliminate bacterial pathogens, reduce inflammation, and promote the healing of the supporting structures of the teeth.^10^ The primary objective of NSPT is the remove supra and subgingival plaque and calculus deposits using handheld instruments such as curettes. However, the efficacy of NSPT in attaining a healthy and stable periodontal status may often be compromised in specific situations, such as in patients with compromised immune systems, as opposed to individuals with systemic health.^21^ Diabetes mellitus (DM) is a chronic metabolic disorder characterised by impaired insulin function, leading to elevated blood glucose levels.^8^ Poorly controlled DM impairs the body’s ability to combat bacterial infections, compromising the integrity of oral tissues, including those in the periodontium.^14,15,20^ Additionally, DM exacerbates inflammation, hindering periodontal and peri-implant healing processes and compromising outcomes of interventions such as NSPT.^20,22,23^ In this regard it is imperative for patients with DM to maintain glycaemic levels (GL) within the normal limits to enhance their immune response and achieve optimal outcomes for therapies including NSPT. Evidence from indexed literature has shown that NSPT in diabetic patients with periodontal disease helps reduce haemoglobin A1c (HbA1c) levels;^4,37^ however, this statement remains a subject of debate.^6,26^

Antimicrobial photodynamic therapy (aPDT) is an innovative approach in the realm of medical and dental treatments, aimed at combating microbial infections.^36^ This technique harnesses the power of light and a photosensitising agent to produce reactive oxygen species which, upon activation by light, induce oxidative damage to microbial cells, effectively eliminating bacteria, fungi, and viruses.^36^ In clinical periodontology and related research, aPDT has gained recognition as a promising adjunct to traditional NSPT, offering a non-invasive and targeted method for eradicating periodontal pathogens.^1,18,19^ In a comprehensive investigation involving 45 patients diagnosed with type-2 DM, Al-Zahrani et al^3^ conducted a study to evaluate the impact of NSPT coupled with adjunct aPDT on GL. Contrary to expectations, the outcomes of this clinical examination revealed that while NSPT with adjunct aPDT effectively reduced periodontal probing depth (PD) and enhanced clinical attachment levels (CAL), it did not yield significant improvements in HbA1c levels within the patient cohort. Interestingly, a randomised controlled trial (RCT) conducted by Macedo Gde et al^24^ presented contrasting findings, demonstrating that NSPT with adjunct intervention did not yield notable enhancements in clinical periodontal parameters but exhibited a significant improvement in GL among individuals diagnosed with type-2 DM. Such controversial outcomes convinced the authors to review pertinent RCTs and format an evidence-based review on the effectiveness of NSPT with adjunct aPDT in reducing HbA1c levels in patients with DM.

The objective of the present systematic review and meta-analysis was to assess the efficacy of NSPT with adjunct aPDT in reducing periodontal inflammation and HbA1c levels in patients with DM.

## MATERIALS AND METHODS

### Ethical Guidelines

The present investigation constitutes an evidence-based review of relevant scientific literature extracted from indexed databases. As such, the study protocol was exempted from the requirement of obtaining prior ethical approval from an institutional review committee and/or board. This decision was rooted in the nature of the research, which involved the examination and synthesis of existing scholarly works and did not involve direct engagement with human subjects.

### Focused Question

The focused question was: ‘Does NSPT with adjunct aPDT help reduce periodontal inflammation and HbA1c levels in patients with DM?’

### Patients, Intervention, Control, Outcome

The PICO was formatted as follows: Patients (P): Participants diagnosed with DM; Intervention (I): NSPT with adjunct aPDT for the treatment of periodontitis; Control (C): NSPT alone or NSPT with adjunct systemic antibiotic therapy; and Outcome (O): Changes in HbA1c levels.

### Inclusion and Exclusion Criteria

In the current investigation, the inclusion criteria comprised of RCTs specifically evaluating the impact of NSPT on HbA1c levels in diabetic patients, with a specific focus on interventions involving NSPT with and without adjunct aPDT. Studies falling under the categories of in-vitro/ex-vivo investigations, case series, case reports, review articles, letters to the Editor, commentaries, expert opinions, and those conducted on animal models were intentionally excluded from the review process.

### Literature Search

The literature search systematically explored the efficacy of NSPT with adjunct aPDT in reducing HbA1c levels in patients diagnosed with DM. The literature search was performed in accordance with the preferred reporting items for systematic reviews and meta-analysis.^30^ The search encompassed the following databases: PubMed/MEDLINE, Cochrane Library, Embase, Web of Science, and Scopus. A combination of medical subject headings (MeSH) terms and keywords related to NSPT, aPDT, DM, and HbA1c was used, employing Boolean operators for refinement. The literature search was performed by one investigator (MSB) without language and time barriers up to and including July 2023. Reference lists of potentially relevant original and review articles were also hand-searched.

### Meta-analysis

To summarise and compare studies, data were displayed as a weighted mean difference in primary outcomes. Using this index, data from articles was directly pooled together (means and 95% CI). For continuous outcomes, mean differences and 95% confidence intervals were used to summarise the data for each study. The study-specific estimates were pooled using the random effects model. Forest plots were created to illustrate the effects of the different studies and the global estimation. Review Manager (RevMan) version 5.3. for Mac from Cochrane collaboration was used to perform all analyses. Statistical significance has been defined as a p value < 0.05.

### Risk of Bias

A standardised approach was employed following the Cochrane risk of bias (RoB) tool.16 The selection process involved identifying eligible RCTs based on predefined inclusion and exclusion criteria as referenced above. Data extraction encompassed critical study details, such as design, participants, interventions, outcomes, and funding sources. Each included study underwent a systematic evaluation across key domains: random sequence generation, allocation concealment, blinding of participants and personnel, blinding of outcome assessment, handling of incomplete outcome data, selective reporting, and identification of other potential biases. One independent investigator (MSB) conducted the risk of bias assessment, with discrepancies resolved through consensus or consultation with a third reviewer if necessary. The overall RoB for each study was categorised as low, unclear, or high, with particular attention to the potential impact on the validity and reliability of the synthesised evidence. This methodology was adopted to ensure a rigorous and transparent evaluation of study quality.

## RESULTS

### General Characteristics of RCTs

**Fig 1:**
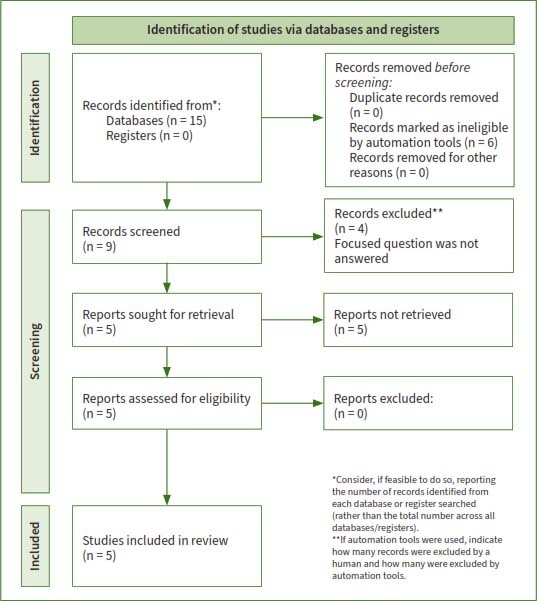
PRISMA flow diagram.

Through the initial search 15 studies were identified. Ten studies, which did not address the focused question were excluded. Five RCTs^3,5,7,24,25^ fulfilled the eligibility criteria and were processed for data extraction (Fig 1). The number of participants ranged from 12 to 45 patients with medically diagnosed type-2 DM. Four RCTs^5,7,24,25^ reported the duration of type-2 DM from at least 5 years to 15.3 ± 5.2 years. In all RCTs,^3,5,7,24,25^ the follow-up duration ranged between 90 and 180 days (Table 1). Prior sample-size estimation was performed in all RCTs.^3,5,7,24,25^

**Table 1:** General characteristics of included randomised controlled trials

Authors	Patients (n)	Mean age	Gender	Type of DM	Duration of DM	Study groups	Follow-up
Al-Zahrani et al^3^	45 patients	52.2 ± 8.35 years	26 males 17 females	Type-2 DM	NR	Group 1: SRP alone Group 2: SRP + doxycycline Group 3: SRP + PDT	90 days
Barbosa et al^5^	12 patients	52.2 years*	4 males 8 females	Type-2 DM	9.58 years*	Group 1: SRP alone Group 2: SRP + PDT	180 days
Brinar et al^7^	24 patients	65.4 ± 4.9 years	13 males 11 females	Type-2 DM	15.3 ± 5.2 years	Group 1: SRP alone Group 2: SRP + PDT	90 days
Macedo Gde et al^24^	30 patients	48.73 ± 7.1 years	NR	Type-2 DM	At least 5 years	Group 1: SRP alone Group 2: SRP + phenothiazine Group 3: SRP + PDT	90 days
Mirza et al^25^	30 patients	51.7 ± 7.63 years	20 males 10 females	Type-2 DM	11.3 ± 2.23 years	Group 1: SRP alone Group 2: SRP + PDT	180 days

*Standard deviation was not reported; DM: diabetes mellitus; SRP: scaling and root planning; PDT: photodynamic therapy.

### Photodynamic Therapy-related Parameters

In all RCTs,^3,5,7,24,25^ aPDT was done using a diode laser with wavelengths ranging between 660 and 810 nm. In three RCTs,^3,5,25^ methylene blue with concentrations ranging between 0.01 and 10 mg/ml. In these studies,^3,5,25^ the duration of photosensitiser application ranged between 10 and 300 seconds and laser irradiation was performed for a duration ranging from 60 to 120 s. In four RCTs,^5,7,24,25^ the diode laser was used at a power ranging between 40 and 250 mW. Barbosa et al.^5^ and Macedo Gde et al.^24^ reported the total output energy, which was 0.8 joules per site and 2.79 joules, respectively. The RCTs by Macedo Gde et al.^24^ and Mirza et al.^25^ reported the energy fluence, which was 16.72 J/cm^2^ and 22 J/cm^2^, respectively. The power density and diameter of fibre tip were reported by one RCT,24 which were 28 mW/cm^2^ and 0.36 mm, respectively (Table 2).

**Table 2:** Photodynamic therapy-related parameters

Authors	Laser	Wavelength	Laser power	Total energy	Energy fluence	Power density	Photosensitiser (concentration)	Duration of photosensitiser application	Duration of irradiation	Diameter of fibre tip
Al-Zahrani et al^3^	Diode	670 nm	NR	NR	NR	NR	Methylene blue (0.01 mg/ml)	10 seconds	60 seconds	NR
Barbosa et al^5^	Diode	660 nm	40 mW	0.8 J/site	NR	NR	Methylene blue (10 mg/ml)	300 seconds	120 seconds	NR
Brinar et al^7^	Diode	810 nm	250 mW	NR	NR	NR	Indocyanine green (1mg/ml)	60 seconds	NR	NR
Macedo Gde et al^24^	Diode	660 nm	60 mW	2.79 J	16.72 J/cm^2^	28 mW/cm^2^	Phenothiazine chloride (10 mg/ml)	60 seconds	10 seconds	0.6 mm
Mirza et al^25^	Diode	670 nm	150 mW	NR	22 J/cm^2^	NR	Methylene blue (0.05 mg/ml)	10 seconds	60 seconds	NR

NR: not reported; J: joules; mg/ml: milligrams per millilitre; mW: milliwatt mW/cm2: milliwatt per square centimetre; mm: millimetres; nm: nanometres.

### Haemoglobin A1c Levels

Four^3,5,7,25^ of the five^3,5,7,24,25^ RCTs reported that NSPT with adjunct aPDT does not influence HbA1c levels.

### Periodontal Inflammation

In two RCTs,^5,24^ NSPT with adjunct aPDT reported no improvement in clinical periodontal parameters. Two studies^3,25^ reported that NSPT with adjunct aPDT significantly reduces periodontal PD compared to NSPT alone. According to Al-Zahrani et al.^3^ and Brinar et al.^7^ NSPT with adjunct aPDT improves CAL and bleeding on probing, respectively. In summary, 60% of the RCTs^5,24,25^ reported that NSPT with adjunct aPDT offers no additional benefits in terms of reduction in periodontal inflammation compared with NSPT alone (Table 3).

**Table 3:** Impact of non-surgical periodontal therapy with and without adjunct antimicrobial photodynamic therapy

Authors	Periodontal status (test-versus control) at follow-up	Test-group (HbA1c levels)		Control-group (HbA1c levels)		Change in HbA1c levels at follow-up		Main outcome
		Baseline	Follow-up	Baseline	Follow-up	Test-group	Control-group	
Al-Zahrani et al^3^	Reduced PD Improved CAL	8.75 ± 1.43%	8.22 ± 0.95%	9.25 ± 2.71%	8.79 ± 2.85%	0.48 ± 0.16%	0.45 ± 0.12%	NSPT+PDT helps reduce periodontal inflammation but not HbA1c levels
Barbosa et al^5^	No improvement in PI, PD, CAL and BoP	8.8 ± 1.7%	9.1 ± 2.0%	7.91 ± 1.9%	7.6 ± 1.7%	0.2 ± 0.05%	0.23 ± 0.03%	NSPT+PDT does not offer additional benefits in terms of reduction in periodontal inflammation NSPT ± PDT does not influence HbA1c levels
Brinar et al^7^	Reduced BoP	7.9 ± 0.3%	7.4 ± 0.2%	8.2 ± 0.3%	7.5 ± 0.2%	0.42 ± 0.12%	0.58 ± 0.12%	NSPT+PDT helps reduce periodontal inflammation but not HbA1c levels
Macedo Gde et al^24^	No improvement in PD, CAL and BoP	8.6 ± 1.1%	7.6 ± 0.6%	8.0 ± 0.9%	7.8 ± 1.3%	0.82 ± 0.15%	0.25 ± 0.11%	NSPT+PDT helps reduce HbA1c levels but not periodontal inflammation
Mirza et al^25^	Reduced PD	7.85 ± 0.21%	7.31 ± 0.19%	7.91 ± 0.34%	7.49 ± 0.26%	0.4 ± 0.05%	0.42 ± 0.11%	NSPT+PDT does not offer additional benefits in terms of reduction in periodontal inflammation NSPT ± PDT does not influence HbA1c levels

BoP: bleeding on probing; CAL: clinical attachment levels; HbA1c: haemoglobin A1c; NSPT: non-surgical periodontal therapy; PD: probing depth; PDT: photodynamic therapy.

### Meta-analysis

**Fig 2:**
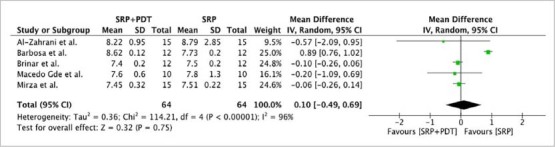
Forest plots demonstrating the comparison of SRP+aPDT versus SRP alone. IV: inverse variance; CI: confidence interval; Total: number of patients.

To assess whether within-study or between-study variability is present, heterogeneity was evaluated. HbA1c levels were evaluated at 90-days follow-up time period for five studies. There was considerable heterogeneity (I2: 96%). Moreover, there was statistically insignificant difference between SRP+PDT and SRP alone with a mean difference of 0.10% (p = 0.75). Figure 2 shows the forest plots for HbA1c levels at 90-days.

### Risk of Bias

**Fig 3:**
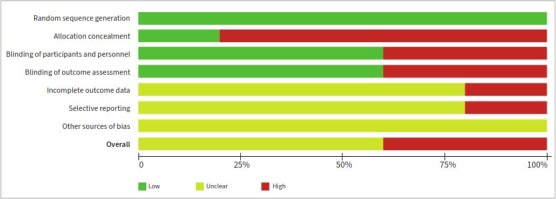
Risk of bias assessment.

Three^5,7,25^ and two^3,24^ RCTs had a moderate and high RoB, respectively (Fig 3).

## DISCUSSION

Optimal glycaemic control plays an essential role in improving the overall wellbeing of patients with metabolic disorders such as DM. Conventionally, dietary control, regular exercise and medications are prescribed to patients for managing their glycaemic status.^28,33^ From an orodental perspective, periodontal and peri-implant inflammation are a common manifestation among diabetic patients particularly among those with a poor metabolic control of DM.^2,9,13,17,29^ The NSPT is the most common procedure performed for the management of periodontal inflammatory conditions such as periodontitis^11^; and it has been reported that this therapeutic protocol also helps reduce HbA1c levels in patients with DM.^32^ Nevertheless, studies^34,35^ have also reported that conventional periodontal treatments such as SRP or NSPT when performed with adjuvant therapies such as aPDT are more effective in reducing periodontal soft-tissue inflammation in contrast to when SRP or NSPT are performed alone. It was therefore anticipated that NSPT with adjuvant aPDT would also contribute towards significantly reducing HbA1c levels in diabetic patients with periodontal diseases in contrast to NSPT alone. A vigilant review of pertinent indexed literature was therefore performed. The author of the current investigation implemented rigorous criteria, specifically limited to the inclusion of RCTs that addressed the focused question. This deliberate selection was undertaken to ensure the acquisition of the utmost level of scientific evidence and to procure the most dependable outcomes for the current review. After a vigilant review of pertinent indexed literature, a limited number of RCTs^3,5,7,24,25^ fulfilled the inclusion criteria and were systematically reviewed. One critical observation regarding the methodology of included RCTs was that the number of included patients was subjected to prior sample-size estimation or power analysis (PA). The PA is a crucial component in scientific research as it helps researchers determine the statistical power of their study, which in turn influences the likelihood of detecting true effects.^12^ Statistical power is the probability that a study will correctly reject a false null hypothesis, or in other words, the probability of avoiding a Type-II error.^12^ Moreover, the test (NSPT+aPDT) and control (NSPT alone) groups were well-defined in all the included RCTs.^3,5,7,24,25^ Notwithstanding the robust methodological foundations, outcomes from 80% of the studies^3,5,7,25^ revealed that the application of NSPT with or without adjunct aPDT did not demonstrate efficacy in ameliorating the glycaemic status of patients diagnosed with type-2 DM.

A number of factors that could have potentially influenced results specifically pertaining to the primary and secondary outcome variable (HbA1c levels and periodontal inflammatory parameters, respectively) reported in these RCTs,^3,5,7,24,25^ should be taken into consideration. In all included RCT^3,5,7,24,25^ aPDT was performed once throughout the study period. In a 6-months’ follow-up clinical investigation, Muzaheed et al.^27^ investigated the antimicrobial effects of single versus multiple sessions of PDT as adjunct to NSPT among patients with periodontitis. The results showed that at least two sessions of aPDT following NSPT are needed to achieve a statistically significant reduction in subgingival bacterial counts in comparison with a single session of aPDT after NSPT.^27^ It is speculated that at least two sessions of aPDT are warranted to significantly reduce periodontal inflammation and improved glycaemic status in patients with type-2 DM. However, according to Ramanauskaite et al.^31^ multiple sessions of aPDT after NSPT do not offer additional benefits in terms of reduction in periodontal inflammation. Further RCTs with long-term follow-up are therefore needed in this regard. It is also noteworthy that the laser parameters and concentrations of photosensitisers used remained inconsistent among these RCTs.^3,5,7,24,25^ For instance, power density were not reported in 80% of the studies^3,5,7,25^ and concentrations of methylene blue ranged between 0.01 and 10 mg/ml in 60%^3,5,25^ of these RCTs. The diameter of a laser fibre tip is a crucial factor in delivering optimal amounts of laser radiation during medical procedures such as laser surgery or laser therapy. The choice of fibre diameter affects various aspects of the laser treatment, including the precision of the procedure, tissue penetration, and overall efficacy. The diameter of the laser fibre tip remained unreported in 80% of the RCTs.^3,5,7,25^ Such limitations seem to have possibly compromised the efficacy of aPDT in terms of reducing periodontal inflammation as well as HbA1c levels in the targeted populations.

It is important to conduct additional RCTs to determine and standardise the optimal laser parameters and photosensitiser concentrations in an attempt to comprehend the precise role of aPDT with adjunct NSPT in the management of periodontal conditions and GL in hyperglycaemic patients.

## CONCLUSION

NSPT with or without adjunct aPDT does not affect HbA1c levels in patients with type-2 DM.
